# Surveillance Snapshot: Non-Hodgkin Lymphoma Incidence in Active Component U.S. Service Members, 2017–2023

**Published:** 2025-02-20

**Authors:** Scott J. Russell, Sithembile L. Mabila

**Affiliations:** 1 Epidemiology and Analysis Branch, Armed Forces Health Surveillance Division, Public Health Directorate, Defense Health Agency, Silver Spring, MD

Lymphomas are defined into 2 categories: Hodgkin lymphomas, which present with Reed-Sternberg cells, and non-Hodgkin lymphomas (NHLs), which do not.^1^ While the narrowly-defined Hodgkin lymphomas, which comprise about 10% of cases, tend to respond well to treatment, the prognoses for NHLs, which account for 90% of lymphomas, vary widely based on a cancer’s subgroup within its greater designation.^2^ Variable treatment successes can be partly explained by difficulties in diagnosis and a wider range of tumor aggression between subtypes.^3^

NHL is 1 of the 10 most diagnosed cancers in the U.S. for both men and women. Generally diagnosed after the age of 60,^3,4,5^ the incidence rate (IR) of all NHL within the U.S. general population in 2021 was 22.1 per 100,000 persons in men and 15.2 in women^6^; for those under age 50 years, rates declined to 5.3 and 3.9, respectively.^7^ A recent study suggests that some cancer rates in military personnel differ from the general population, but no prior analyses nor determinations of historical rates of NHL within the U.S. military population exist.^7^

This analysis utilized an updated case definition developed by the Armed Forces Health Surveillance Division (AFHSD), based on consultation with subject matter experts and previous literature, which divides the International Classification of Diseases, 10th Revision (ICD-10) codes for NHL into 6 subgroups.^8^ Follicular, non-follicular, and mature T/NK cell lymphomas (**Table**) refer to specific cancer subgroups, while the other cancer types denote broader subgroup categories.^8^ These definitions were applied to the data in the Defense Medical Surveillance System (DMSS)’s inpatient and outpatient records from January 2017 through December 2023 for active component service members (ACSMs). An incident case was defined as 1 qualifying inpatient diagnosis in the first diagnostic position, a diagnosis in the second diagnostic position with a qualifying treatment code in the first diagnostic position, or 3 outpatient visits with qualifying diagnoses within 90 days of one another.^8^ Only the first lifetime diagnosis was considered incident. The total person time for all eligible ACSMs was then calculated to define the incidence rates for each subgroup (**Table**).

A total of 621 incident cases in this study contributed to the overall IR of 6.6 cases per 100,000 person-years (p-yrs). The number and IR were higher among men (n=535, IR 6.8 per 100,000 p-yrs) compared to women (n=86, IR 5.39 per 100,000 p-yrs), and a majority of men (n=327) were of non-Hispanic White race or ethnicity (data not shown). These results are consistent with the population distribution of the U.S. military, which is majority non-Hispanic White male, and do not suggest any race-based effects on lymphoma diagnosis.

Specified and Unspecified NHL had the highest overall IR (2.6 per 100,000 p-yrs) over the surveillance period (**Figure**). There is a modest increase in IR, especially among Specified and Unspecified NHL diagnoses over the 7-year surveillance period (**Figure**). These rates are far lower than the non-age stratified reported national rates—19.0 per 100,000 for men and 15.8 for women—because the military population is much younger, with most cases occurring between ages 20 and 45 years, with only 1 in the older than age 60 years demographic (data not shown). Overall, lymphoma rates were low among ACSMs during the surveillance period.

## Figures and Tables

**Table:** Lymphoma Subtype Rates^a^ Among U.S. Active Component Service Members, 2017-2023

Cancer Type	**2017**		**2018**		**2019**		**2020**		**2021**		**2022**		**2023**	
	No.	IR	No.	IR	No.	IR	No.	IR	No.	IR	No.	IR	No.	IR
Specified and unspecified NHL	40	3.0	34	2.5	30	2.2	29	2.1	30	2.2	38	2.8	43	3.3
Non-follicular	33	2.5	29	2.2	24	1.8	21	1.5	16	1.2	28	2.1	18	1.4
Mature T/NK cell	9	0.7	13	1.0	9	0.7	8	0.6	17	1.2	14	1.0	14	1.1
Follicular	10	0.8	8	0.6	16	1.2	16	1.2	11	0.8	9	0.7	9	0.7
Malignant proliferative/B cell	7	0.5	6	0.4	8	0.6	3	0.2	5	0.4	2	0.1	5	0.4
Other specified T/NK cell	1	0.1	3	0.2	1	0.1	0	0.0	3	0.2	1	0.1	0	0.0

Abbreviations: No., number; IR, incidence rate; NHL, non-Hodgkin lymphoma.

^a^Rates per 10,000 person-years.

**Figure:**
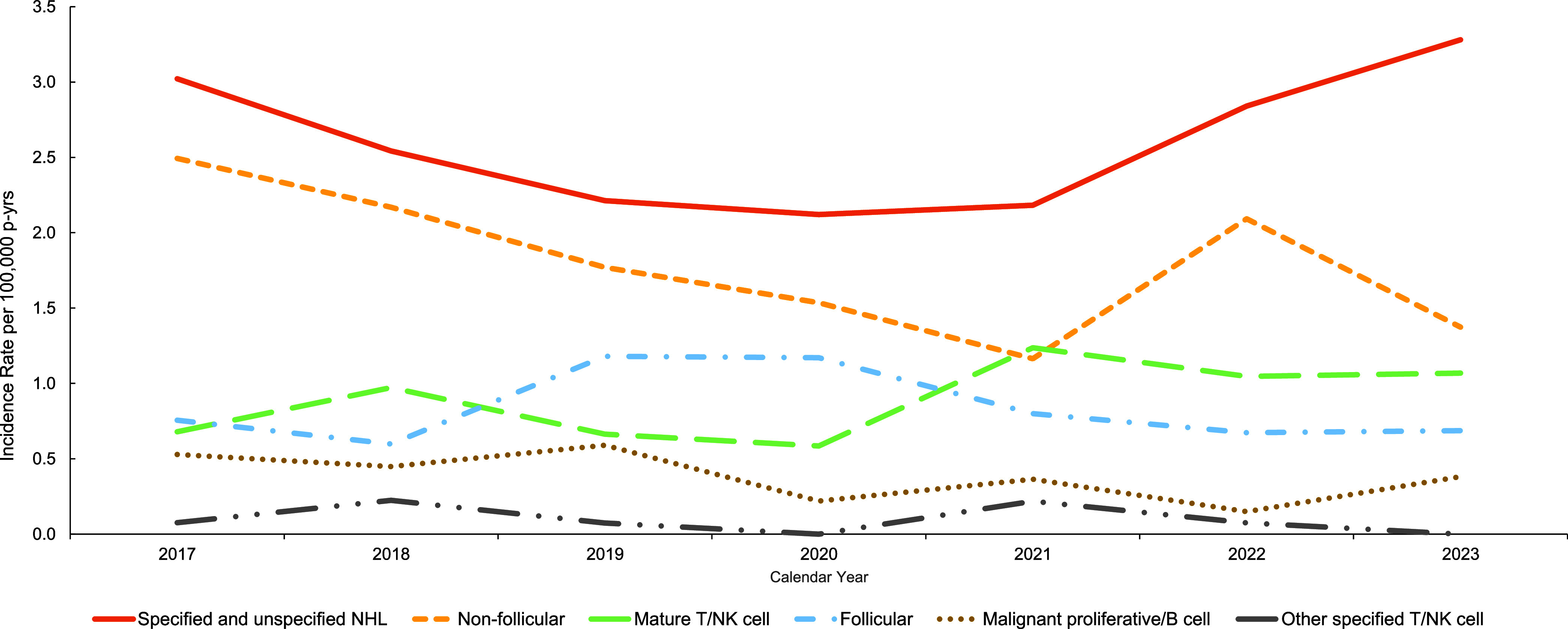
Non-Hodgkin Lymphoma Subtype and Overall Rates Among Active Component U.S. Service Members, 2017-2023

## AUTHORS' AFFILIATION

Epidemiology and Analysis Branch, Armed Forces Health Surveillance Division, Public Health Directorate, Defense Health Agency, Silver Spring, MD
